# Effects of temperature, pressure, and hydration on the microstructural characteristics and mechanical properties of calcite

**DOI:** 10.3389/fchem.2025.1568585

**Published:** 2025-06-25

**Authors:** Xiaoyi Zhou, Qiuqi Chen, Xin Tang, Ruiyu He, Zhangping Yan, Junjie Xiong, Yuhang Zhou, Linyan Li

**Affiliations:** ^1^ School of Civil Engineering, Chongqing Three Gorges University, Chongqing City, China; ^2^ School of Resources and Earth Sciences, China University of Mining and Technology, Xuzhou City, Jiangsu, China

**Keywords:** calcite, hydration, molecular dynamics simulation, lattice parameter, elastic modulus

## Abstract

Geotechnical geological disasters occur frequently in China. Especially under complex environmental conditions, the failure mode of rock and the change mechanism of its mechanical properties are not clear. Although some progress has been made in recent studies on the physical and chemical properties of rocks and the microscopic mechanism of action, there is still a lack of systematic understanding of the change mechanism of calcite under different temperature, pressure and humidity conditions. In this paper, the influence of these environmental factors on the expansion behavior and elastic modulus of rock is deeply analyzed by constructing a calcite supercell model. The results show that the lattice parameters a, b and c of calcite increase by 0.45%, 0.45% and 0.44%, respectively, when the temperature increases from 300 K to 1000 K. At the same time, the bulk modulus, shear modulus and Young‘s modulus decreased by 6.45%, 3.63% and 3.92%, respectively. When the pressure increases from 0.1 GPa to 0.5 GPa, the volume of calcite crystal decreases by 1.10%, while the bulk modulus, shear modulus and Young’s modulus increase by 2.74%, 9.36% and 8.66%, respectively. The bulk modulus, shear modulus and Young‘s modulus decreased by 15.6%, 18.5% and 18.1%, respectively, when anhydrous calcite was transformed into 50 water molecules. This study clarifies the degradation mechanism of calcite under the action of temperature, pressure and hydration, and provides an important theoretical basis and guidance for the prevention and control of geotechnical geological disasters.

## 1 Introduction

Geotechnical engineering is involved in civil engineering, water conservancy, highways, bridges, tunnels, and oil and gas mining. During production, the complicated geological environment often induces changes in the microstructure and mechanical properties of rocks. This leads to frequent engineering disasters (Zhang et al., 2024; Chen and Xu, 2024; Cai et al., 2024; Sarker and Karim, 2023). While current research has advanced our understanding of rock deterioration and mechanical behavior, it remains largely focused on macroscopic testing and support technologies, leaving a gap in fundamental microstructural analysis (Hassanain and Albugami, 2024; Xing and Xing, 2020; Dong et al., 2024; Wang et al., 2020)^]^. Calcite, a major constituent of carbonate rocks and particularly abundant in dolomitic formations, is of particular interest due to its engineering relevance and sensitivity to environmental changes. Understanding how calcite behaves under varying conditions of temperature, pressure, and hydration is crucial for infrastructure safety and disaster risk mitigation (Kushnir et al., 2015; Tang et al., 2020; Mijowska et al., 2020).

Effenberger et al. (1981) used XRD (X-ray diffraction) to refine the single-crystal structure of calcite group minerals. They obtained accurate data, including lattice parameters and diffraction intensities, and predicted the lattice parameters of calcite. Rao et al. (1968) investigated the crystal structure and thermomechanical properties of calcite, revealing that warming would lead to a slight expansion of calcite’s crystal volume. Jiang et al. (2019) combined triaxial testing with CT (computed tomography) scanning technology to monitor the mechanical damage process of rocks in real time, elucidating the rocks’ response to stress. Additionally, Sun et al. (2020) utilized advanced techniques such as nuclear magnetic resonance (NMR) and electron microscope imaging (EMMI) to analyze the microstructural changes and macrodeformation characteristics of shale after prolonged immersion in various liquids.

These studies confirm that calcite’s physicochemical behavior is highly sensitive to temperature, pressure, and water content. However, most of this research relies heavily on physical experimentation, which has limitations in capturing atomistic mechanisms and long-term environmental effects (Hashim and Kaczmarek, 2020; Koga et al., 2016; Ruiz-Agudo et al., 2015). As a result, the internal mechanisms governing microstructural and mechanical evolution under complex environmental conditions remain insufficiently understood. To address these limitations, molecular dynamics (MD) simulation has emerged as a promising method for revealing nanoscale interactions under complex conditions. Recent studies have demonstrated the value of MD in exploring the influence of environmental factors on mineral behavior, including hydration, thermal expansion, and stress response (Kerisit and Prange, 2020; Wang et al., 2023; Ali et al., 2020; Lammers et al., 2020). Despite these advances, the application of MD simulations specifically to calcite under combined temperature, pressure, and hydration conditions remains limited. (Rukuan et al., 2020; Jiamin, 2021; Liu et al., 2021).

Previous studies have explored the thermal and mechanical behavior of calcite and other carbonate minerals under specific environmental conditions; however, these investigations often focus on limited variables or lack atomic-scale insight into combined temperature, pressure, and humidity effects. In particular, there remains a gap in understanding how calcite’s microstructure and mechanical properties evolve simultaneously under multiple environmental influences—especially using molecular dynamics (MD) simulations. This study deepened the understanding of the microstructural characteristics and mechanical properties of calcite under different temperature, pressure and humidity conditions. By employing molecular dynamics simulations, we aim to bridge this gap by systematically analyzing the behavior of calcite across varying environmental parameters and revealing the underlying mechanisms driving its structural and mechanical responses.

The insights gained from this research provide essential technical support and a theoretical basis for civil engineering, enhancing existing design methods and improving infrastructure resilience against natural disasters. The application of molecular simulation offers refined tools for understanding the physical and chemical properties of carbonate rocks, which is crucial for engineering decision-making, disaster risk management, and resource exploitation in weak geological environments.

Furthermore, this research not only guides future studies on dolomite and similar materials but also promotes interdisciplinary collaboration and inspires innovation in engineering practices. Ultimately, it contributes to safer and more sustainable civil infrastructure by improving material selection and design, enhancing risk assessments, and informing effective disaster mitigation strategies.

## 2 Molecular dynamics simulation

LAMMPS, developed by Sandia National Laboratories (SNL), is an open-source and powerful software used for constructing complex systems for adsorption and molecular dynamics simulations (Pal and Reddy, 2024; Guo et al., 2018; Humbert et al., 2019). It is typically necessary to combine it with OVITO software for model processing and data analysis (Gissinger et al., 2024). Therefore, the molecular simulation, model processing, and data analysis discussed in this paper are carried out using these two software packages.

### 2.1 Model construction

**FIGURE 1:**
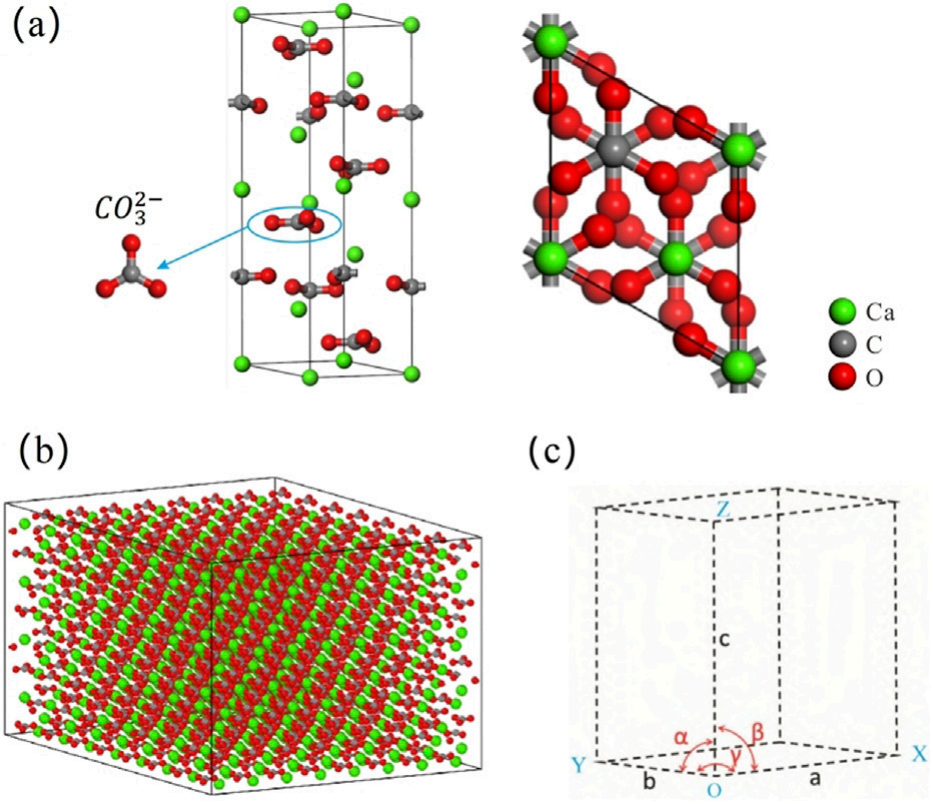
Calcite cell model and schematic diagram of structural parameters **(a)** Crystal cell model; **(b)** Supercell cell model; **(c)** The schematic diagram of lattice parameters.

Calcite is a crystalline structure of calcium carbonate and the most common form of natural calcium carbonate. It is widely distributed in nature, with the chemical formula CaCO_3_. Calcite belongs to the trigonal crystal system and R-3c space group (Karunadasa et al., 2019). The calcite data in this paper comes from American Mineralogist Crystal Structure Database, which was obtained by Zhao Y et al.using XRD and other experiments. Based on this, the initial model of calcite crystal cell was successfully constructed (Zhao et al., 2024) (Figure 1a). The lattice parameters are α = γ = 90°, β = 120°, a = 4.980 Å, b = 4.980 Å, and c = 17.192 Å. The single crystal cell contains 30 atoms and has a density of 2.71 g⋅cm^−3^ (Table 1). To increase the accuracy of the molecular dynamics simulation, the model was scaled separately in the x, y, and z directions using an 8a × 8b × 2c supercell expansion to create a calcite model containing 3840 atoms (Figure 1b) with a cell size of 39.840 Å × 39.840 Å × 34.384 Å (Figure 1c). We pay special attention to maintaining the crystal orientation during the expansion process and ensure that the interatomic interactions in the extended supercell remain effective. This method allows more accurate representation of material properties due to the increase in the number of atoms, thereby better sampling the configuration space.

**TABLE 1:** Atomic coordinates of calcite crystal cell.

Element	X	Y	Z
Ca	0	0	0
C	0	0	0.25
O	0.2578	0	0.25

**FIGURE 2:**
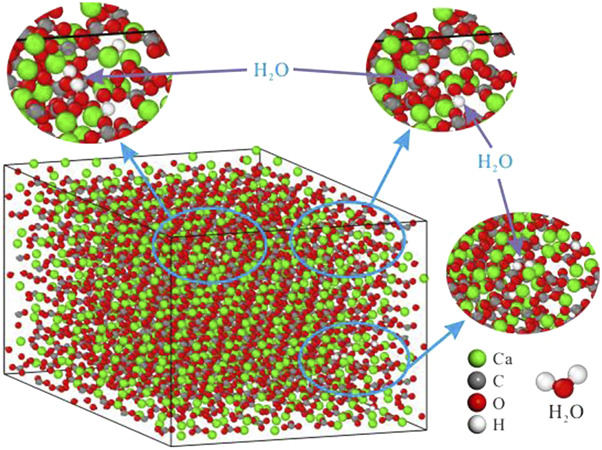
Structure diagram of hydrated calcite model.

Additionally, a small number of water molecules were gradually added to the calcite supercells using the Monte Carlo method (Table 2) to construct the calcite hydration model under different water contents (Figure 2). Monte Carlo method is a statistical simulation method, which is widely used to deal with the randomness and thermodynamic characteristics of complex systems. In this study, we used this method to ensure that a uniform and random distribution can be generated when water molecules are inserted into the calcite supercell, reflecting the interaction between water molecules and calcite crystals in the real environment.

**TABLE 2:** The water content of calcite under different degrees of hydration.

Number of water molecules/unit	0	10	20	30	40	50
water content/%	0	0.073	0.146	0.218	0.291	0.364

Specifically, we set different numbers of water molecules (as shown in Table 2), randomly select the position of the water molecule each time and verify its distance from the atoms in the calcite structure to ensure that no overlap or unreasonable distribution is caused.

This process is continuously iterated until the preset degree of hydration is reached, so that the obtained hydration model can accurately simulate the calcite environment under different water contents. In this way, we construct the hydration model of calcite under different hydration states.

### 2.2 Simulation details

**FIGURE 3:**
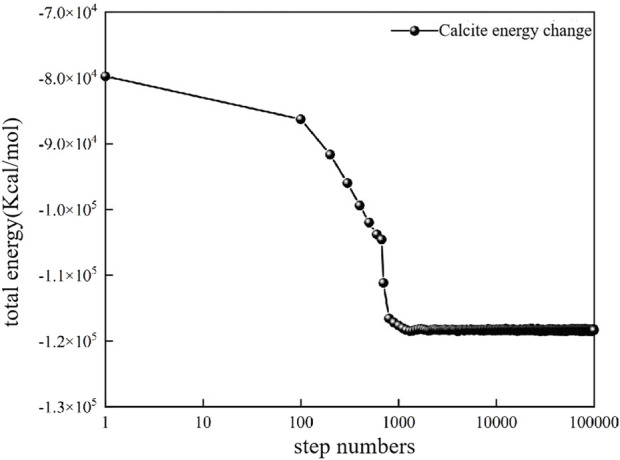
Total energy change in the process of structural optimization of calcite supercell model.

**FIGURE 4:**
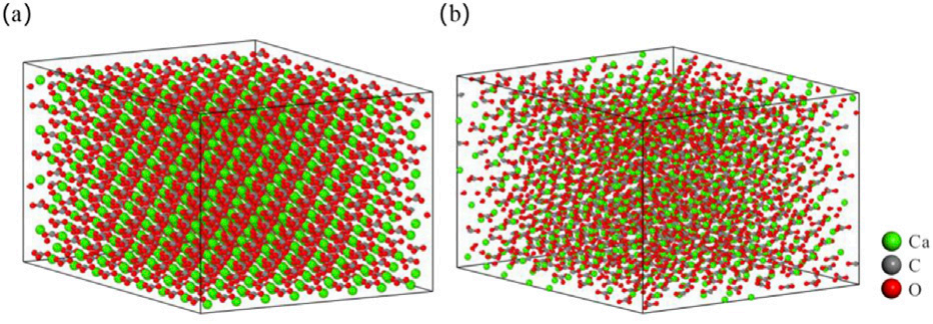
Structure diagram of calcite supercell model. **(a)** Before structural optimization; **(b)** After structural optimization.

In order to ensure the reliability and stability of the structure, the energy of the molecular model is reduced by adjusting the structure configuration. During the whole optimization process, the total energy decreased from −79721.156 to −118371.75 kcal·mol^-1^ (Figure 3). When the calculation step reaches 1,000, the energy of the model tends to be stable, and the molecular model is the configuration of the subsequent molecular dynamics simulation (Figure 4).

In this study, only the effect of a single variable on the microstructure and mechanical properties of calcite was considered. The system pressure was set at standard atmospheric pressure (101 kPa). The temperature was increased from 300 K to 1000 K in 100 K intervals, resulting in a total of eight temperature gradients. This approach enabled the analysis of microcrystal structure and mechanical property changes in calcite at different temperatures. The system was maintained at a standard temperature (300 K), and pressure was gradually increased from 0.1 GPa to 0.5 GPa in intervals of 0.1 GPa, resulting in five pressure gradients. This setup was used to examine the mechanical properties of calcite under different pressure environments. Additionally, the constructed calcite hydration model will undergo molecular dynamics simulation at room temperature and pressure (300 K and 101 kPa) to analyze the diffusion, distribution, and interaction of water molecules in calcite. This analysis aims to explore the microstructural characteristics and mechanical properties of calcite under hydration (details can be found in the Supplementary Material).

## 3 Simulation results and analysis

### 3.1 Microstructural characteristics

**FIGURE 5:**
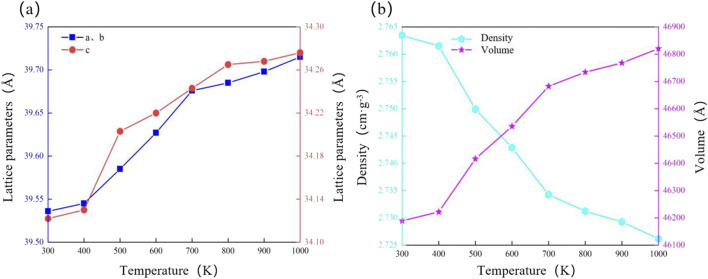
Reaction mechanism of calcite microstructure to temperature. **(a)** Lattice parameter; **(b)** Density and volume.

The lattice parameter and volume of calcite increase with rising temperature, while the density decreases. Calcite undergoes thermal expansion, meaning that interatomic distances increase as temperature rises, causing the crystal structure to enlarge. This expansion occurs due to increased thermal motion and vibration of the atoms, which overcome interatomic forces, resulting in an expanded crystal lattice. Notably, the thermal expansion of calcite is anisotropic; it varies with the orientation of the crystal axes. A detailed analysis of the calcite data reveals that the lattice parameter a, b, and c increase by 0.45%, 0.45%, and 0.44%, respectively. Yoshikazu Suzuki reported anisotropic thermal expansion in calcite, with the a-axis expanding by 0.43% and the c-axis by 0.41% from 298 K to 700 K, attributed to the weaker bonding along the a-axis in the carbonate groups (Suzuki and Ohji, 2004). TIn our simulation, the lattice parameters a, b, and c increased by 0.45%, 0.45%, and 0.44%, respectively, as the temperature rose from 300 K to 1000 K. The overall volume expanded by 1.37%, and the density decreased by 1.35% (Figure 5; Table 3). These results are consistent with the general thermal behavior of calcite reported in previous studies (Zhu et al., 2022; Moslehy et al., 2022; Zhu et al., 2021). Supporting the validity of our simulation approach.

**TABLE 3:** Physical parameters of calcite under different temperature environments.

Temperature (K)	A(Å)	b(Å)	c(Å)	V (Å^3^)	Density (g·cm^−3^)
300	39.536	39.536	34.122	46189.3	2.763
400	39.545	39.545	34.130	46221.9	2.762
500	39.585	39.585	34.203	46416.4	2.750
600	39.627	39.627	34.220	46535.7	2.743
700	39.676	39.676	34.243	46682.6	2.734
800	39.685	39.685	34.265	46734.1	2.731
900	39.698	39.698	34.268	46767.9	2.729
1,000	39.715	39.715	34.274	46820.7	2.726

**FIGURE 6:**
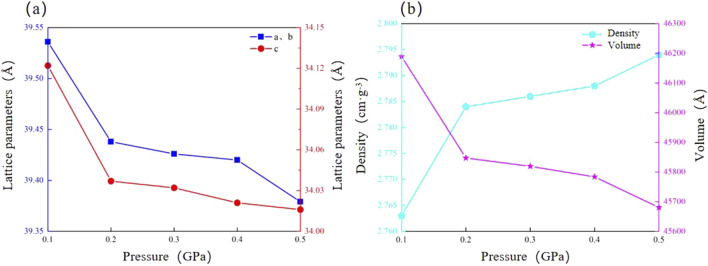
Reaction mechanism of calcite microstructure to pressure. **(a)** Lattice parameter; **(b)** Density and volume.

The simulation results indicate that calcite’s microstructure responds to pressure and temperature through opposite mechanisms. Under high pressure, the crystal structure of calcite compresses and densifies. This compression results from reduced interatomic distances and angles, which are influenced by external pressure. During the pressure increase from 0.1 GPa to 0.5 GPa, the calcite lattice parameter a, b, and c decreased by 0.39%, 0.39%, and 0.31%, respectively. The volume decreases by 1.10%, while the density increases by 1.11% (Figure 6; Table 4). The a- and b-axes, parallel to the basal plane of the calcite structure, are more compressed than the c-axis, which is perpendicular to the basal plane. This is consistent with the experimental results of calcite by Ishizawa, Li Z and Xu B et al. (Ishizawa et al., 2013; Li et al., 2017; Xu and Poduska, 2014).

**TABLE 4:** Physical parameters of calcite under different pressure environments.

Pressure (GPa)	a (Å)	b (Å)	c (Å)	V (Å^3^)	Density (g·cm^-3^)
0.1	39.536	39.536	34.122	46189.3	2.763
0.2	39.438	39.438	34.037	45847.4	2.784
0.3	39.426	39.426	34.032	45819.2	2.786
0.4	39.420	39.420	34.021	45783.9	2.788
0.5	39.379	39.379	34.016	45680.7	2.794

**FIGURE 7:**
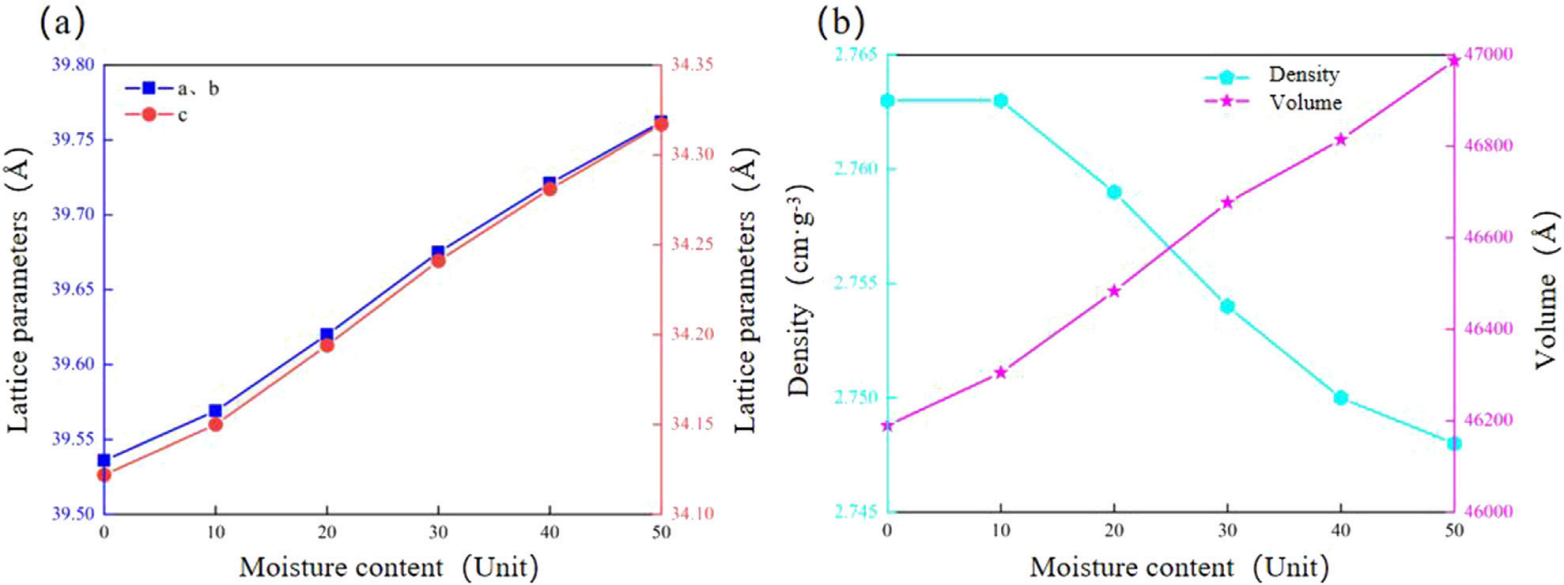
Reaction mechanism of calcite microstructure to hydration degree. **(a)** Lattice parameter; **(b)** Density and volume.

With increasing hydration, the lattice parameters of calcite show varied trends. Both a and b exhibit increases in lattice parameters from 39.536 Å to 39.762 Å, while c increases from 34.122 Å to 34.317 Å, both showing an increase of 0.21%. Moreover, the crystal volume significantly increases from 46189.3 Å^3^ to 46987.6 Å^3^, reflecting an increase of 0.64%. The crystal density decreases by 0.47%, from 2.763 g·cm^-3^ to 2.748 g·cm^-3^ (Figure 7; Table 5). These results suggest that hydration causes microexpansion of the calcite crystal structure, increasing pore size and decreasing density (Chi et al., 2023). The simulation results are consistent with the research results of calcite by Shanmugasundaram et al. (2024), Han et al. (2024). Minor differences in the magnitude of thermal expansion may stem from variations in simulation parameters, such as the applied temperature range, boundary conditions, and interatomic potential functions. In particular, we employed the force field developed by Dove et al. (1992), which was selected for its accuracy in modeling calcite structures under thermal stress. However, other studies, such as those using flexible SPC (Simple Point Charge) models to represent water-calcite interactions (Perry et al., 2007). may report slightly different thermal responses due to differing treatment of water molecules and interaction potentials.

**TABLE 5:** Lattice parameters of calcite under different hydration environments.

Water molecules Quantity (number)	a (Å)	b (Å)	c (Å)	V (Å^3^)	Density (g·cm^−3^)
0	39.536	39.536	34.122	46189.3	2.763
10	39.569	39.569	34.150	46305.2	2.763
20	39.620	39.620	34.194	46483.1	2.759
30	39.675	39.675	34.241	46677.1	2.754
40	39.721	39.721	34.281	46814.5	2.750
50	39.762	39.762	34.317	46987.6	2.748

These methodological distinctions offer insight into both the consistencies and the deviations observed across studies and emphasize the importance of force field selection and system setup in accurately predicting the thermomechanical response of calcite.

**FIGURE 8:**
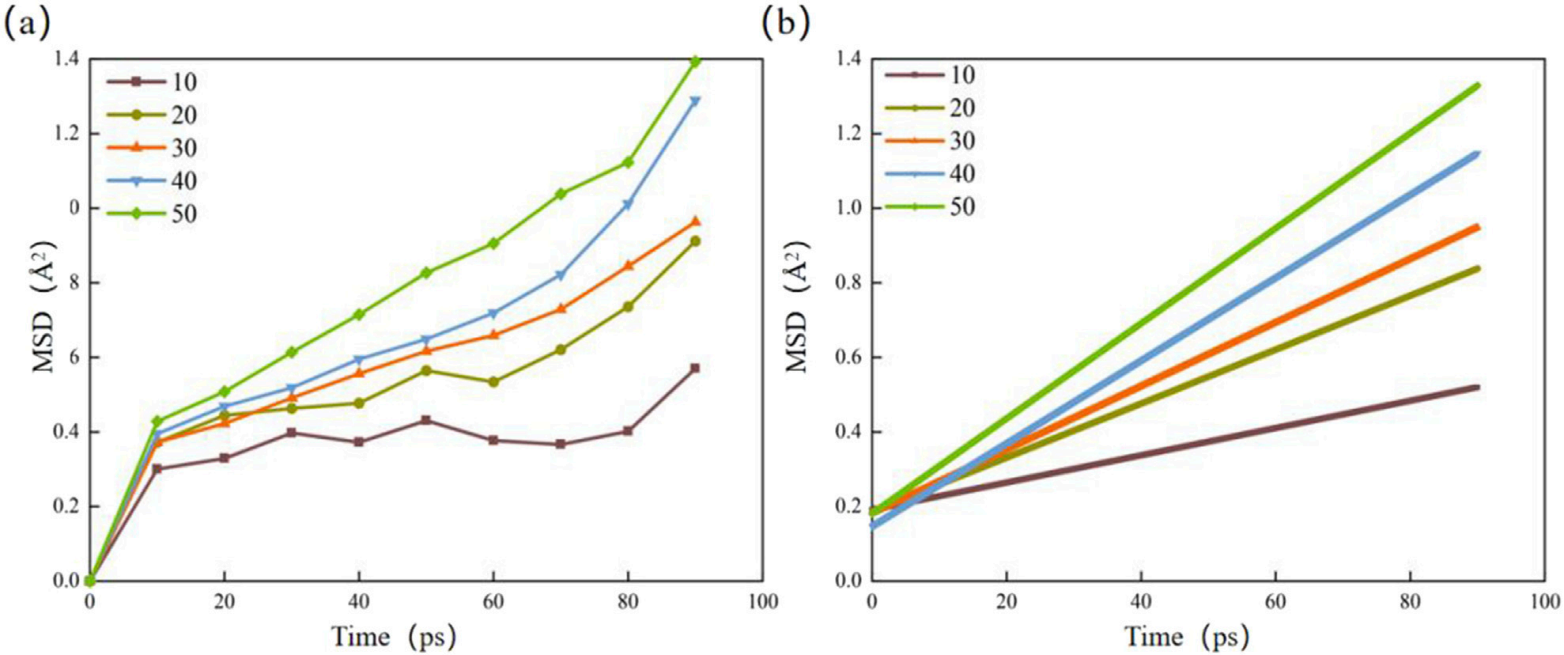
Mean square displacement and linear fitting of water molecules under different hydration degrees. **(a)** Mean square displacement; **(b)** Linear fitting.

The MSD was calculated from 100 ps of hydrated calcite simulated under the NPT ensemble, yielding the diffusion coefficients of interlayer water molecules (Table 6; Figure 8). The results show that the diffusion coefficient of water molecules in hydrated calcite ranges between 6.083 × 10^−12^ ∼ 2.125 × 10^−10^ m^2^/s. The diffusion coefficient tends to increase with greater hydration levels. This trend may result from an increase in water molecules, leading to greater spacing between calcite layers. Consequently, the non-bonding forces between the upper and lower calcite lamellae are weakened, reducing the binding forces of water molecules on the calcite mineral’s surface (Tse, 2020).

**TABLE 6:** Diffusion coefficients of water molecules under different hydration degrees.

Number of water molecules (unit)	The slope of fitting	Diffusion coefficient (Å^2^·ps^−1^)	Diffusion coefficient (m^2^·s^−1^)
10	0.00365	6.083 × 10^−4^	6.083 × 10^−12^
20	0.00723	1.205 × 10^−3^	1.205 × 10^−11^
30	0.0582	9.700 × 10^−3^	9.700 × 10^−11^
40	0.1112	1.853 × 10^−2^	1.853 × 10^−10^
50	0.1275	2.125 × 10^−2^	2.125 × 10^−10^

Compared with the simulation and experimental results in other literatures, some of them have studied the diffusion behavior of water molecules in different minerals by molecular dynamics simulation. For example, Yu et al. (2023) studied the diffusion coefficients of water molecules in illite with different hydration degrees, and found that the diffusion coefficients ranged from 0.35 × 10^−10^ to 7.81 × 10^−10^ m^2^/s. Ma (Marry et al., 2008) studied the diffusion coefficient of water molecules in montmorillonite with different hydration degrees, and found that it ranged from 0.63 × 10^−10^ to 6.77 × 10^−10^ m^2^/s. The result of the study showed that the deeper the hydration degree of illite or montmorillonite, the greater the diffusion coefficient of water molecules. Marry et al. (2002) simulated the diffusion coefficient of water molecules in hydrated montmorillonite, and obtained a value of about 3.9 × 10 ^−10^ m^2^/s. The research results are consistent with the findings of this paper, which further proves the rationality of the simulation.

Concentration distribution curves are a useful tool for studying the structural information of water molecule-calcite interactions. These curves illustrate the ratio of the density of particle A in a specific thickness interval in the normal direction to the total density in the system (Zhang, 2011). Based on the concentration distribution of water molecules in the calcite interlayer, the distribution of water molecules under varying degrees of hydration was analyzed. This analysis aimed to clarify the adsorption characteristics of water molecules on calcite.

**FIGURE 9:**
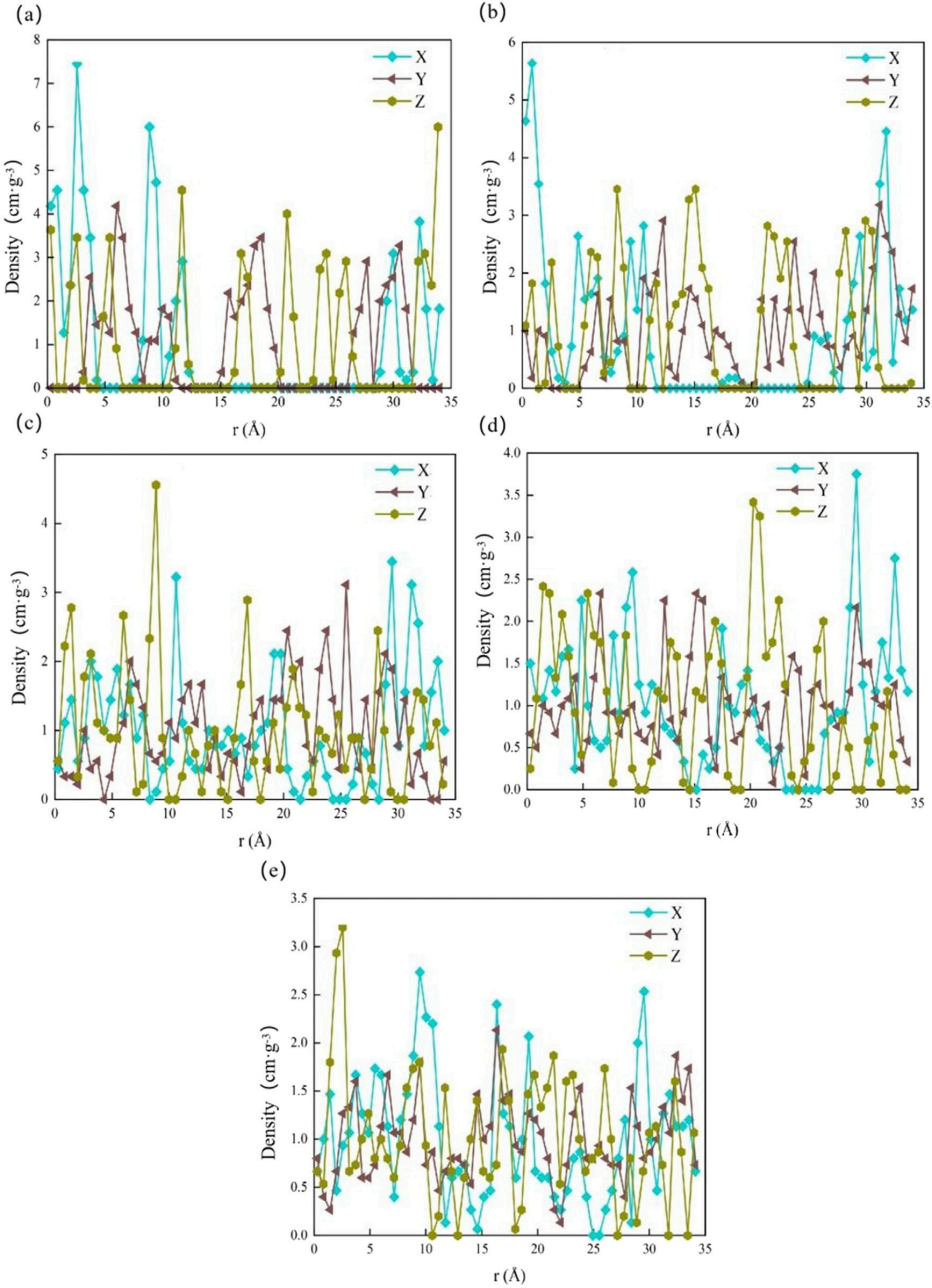
Concentration distribution of water molecules in calcite under different hydration degrees. **(a)** 10 water molecules; **(b)** 20 water molecules; **(c)** 30 water molecules; **(d)** 40 water molecules; **(e)** 50 water molecules.

The simulation results indicate that water molecules are uniformly distributed in the Y and Z directions when the calcite supercells contain 10 and 20 water molecules. However, a significant displacement of water molecules toward the center of the cell occurs in the X direction when the interlayer contains 20 water molecules (Figures 9a,b). As hydration increases, the water molecules in the X, Y, and Z directions gradually achieve a more uniform distribution. This distribution leads to the gradual penetration of hydration into the crystal interior, transitioning from weak adsorption in the X direction of calcite (Figures 9c–e). Additionally, the diffusion coefficient increases as hydration intensifies. This increased water density accelerates the linear expansion of the crystals, enlarges the layer spacing, and exacerbates the deterioration effect on calcite (Wang et al., 2021). Over extended periods, such hydration-induced expansion and interlayer weakening may significantly compromise the structural integrity of calcite-bearing rocks, particularly in groundwater-rich environments. The simulation results are consistent with the research results of Jiang W et al. (Jiang et al., 2024). These findings have important implications for infrastructure projects such as tunnels, foundations, and slope stabilization systems in regions subject to groundwater infiltration. The progressive degradation of calcite through hydration could lead to reduced load-bearing capacity and increased susceptibility to failure, highlighting the need for material durability assessments in the planning and maintenance of civil structures.

### 3.2 Mechanical properties characterization

**FIGURE 10:**
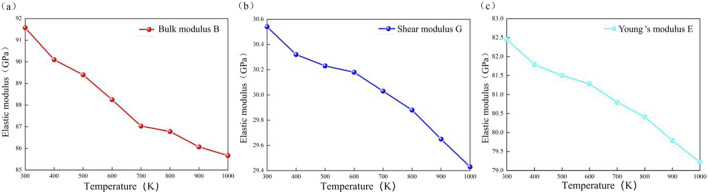
Reaction mechanism of elastic modulus of calcite to temperature. **(a)** Bulk modulus; **(b)** Shear modulus; **(c)** Young’s modulus.

The elastic modulus of calcite was calculated under various factors, based on the stiffness and flexibility matrices obtained from molecular dynamics simulations. The results demonstrate that the elastic modulus of calcite is linearly correlated with temperature changes. As temperature increases, the elastic modulus of calcite gradually decreases, along with its hardness. Specifically, as the temperature rises from 300 K to 1000 K, the bulk modulus decreases from 91.58 GPa to 85.67 GPa, a reduction of 6.45%. The shear modulus decreased by 3.63% from 30.54 GPa to 29.43 GPa, and the Young’s modulus decreased by 3.92% from 82.45 GPa to 79.22 GPa (Figure 10; Table 7). Among these, the change in bulk modulus is the most pronounced. The trends in elastic modulus changes align with those in microstructural characteristics, indicating that as temperature increases, the lattice parameters and volume of calcite increase, while density and strength decrease (Jiamin, 2021; Zhu et al., 2022). This temperature-dependent behavior has critical implications for geotechnical applications. For instance, in deep geological environments with elevated geothermal gradients (e.g., underground tunnels or hydrocarbon reservoirs), the reduced stiffness and strength of calcite-bearing rocks may compromise slope stability or increase deformation risks in tunnel walls. Conversely, during rapid cooling events (e.g., hydraulic fracturing or CO_2_ injection), thermally induced stress concentrations could promote fracture propagation in calcite-cemented formations The simulation results are consistent with the research results of Wa et al. (2025), Luo et al. (2022). Furthermore, in pressure-sensitive environments such as deep geological formations, changes in elastic properties may alter stress distribution and deformation behavior, potentially increasing the risk of structural failure or instability in rock slopes and underground excavations. This emphasizes the importance of accounting for thermomechanical behavior of calcite in engineering designs for infrastructure durability and safety.

**TABLE 7:** The bulk modulus B (GPa), shear modulus G (GPa), Young’s modulus E (GPa), Poisson’s ratio μ parameter of calcite at different temperatures.

Temperature (K)	Bulk modulus B (GPa)	Shear modulus G (GPa)	Young‘s modulus E (GPa)	Poisson’s ratio μ
300	91.58	30.54	82.45	0.350
400	90.10	30.32	81.79	0.349
500	89.40	30.23	81.50	0.348
600	88.25	30.18	81.28	0.347
700	87.03	30.03	80.80	0.345
800	86.78	29.88	80.41	0.346
900	86.07	29.65	79.79	0.345
1,000	85.67	29.43	79.22	0.346

**FIGURE 11:**
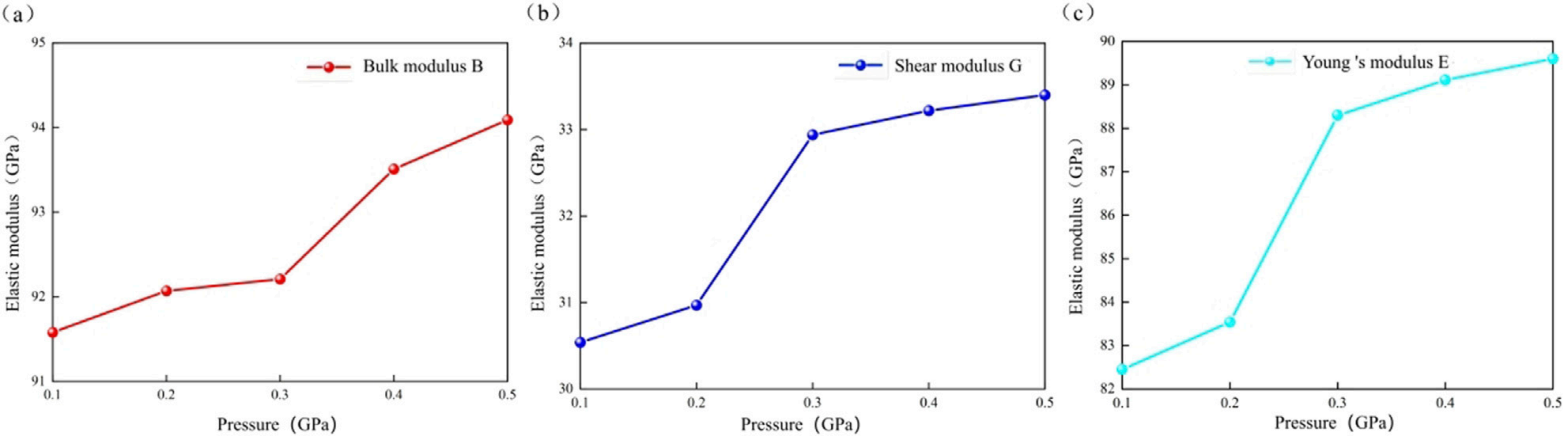
Reaction mechanism of elastic modulus of calcite to pressure. **(a)** Bulk modulus; **(b)** Shear modulus; **(c)** Young’s modulus.

The modulus of elasticity of calcite increases linearly with increasing pressure. When the pressure is increased from 0.1 GPa to 0.5 GPa, the bulk modulus increases from 91.58 GPa to 94.09 GPa, an increase of 2.74%. The shear modulus increased by 9.36% from 30.54 GPa to 33.40 GPa, while the Young’s modulus increased by 8.66% from 82.45 GPa to 89.60 GPa (Figure 11; Table 8). The results are consistent with those of Sedgh et al. (2016), Silvestri et al. (2019). This is because the high-pressure environment contributes to making calcite harder, enhancing its ability to resist volume deformation and shear damage. This hardening behavior under pressure shares similarities with transition metal borides like TiB and ZrB_2_, where high-pressure compression enhances covalent bonding strength (Kang et al., 2024; Jin et al., 2022). The increase in the elastic modulus of calcite with pressure is likely due to the compression of calcite crystals, which decreases lattice parameters and interatomic distances. This increase in interatomic forces and bond strengths reduces susceptibility to deformation and enhances the elastic modulus of calcite (Jiamin, 2021; Ishizawa et al., 2013).

**TABLE 8:** The bulk modulus B (GPa), shear modulus G (GPa), Young’s modulus E (GPa), Poisson‘s ratio μ parameter of calcite under different pressures.

Pressures (GPa)	Bulk modulus B (GPa)	Shear modulus G (GPa)	Young‘s modulus E (GPa)	Poisson’s ratioμ
0.1	91.58	30.54	82.45	0.350
0.2	92.07	30.97	83.54	0.350
0.3	92.21	32.94	88.30	0.340
0.4	93.51	33.22	89.11	0.341
0.5	94.09	33.40	89.60	0.341

**FIGURE 12:**
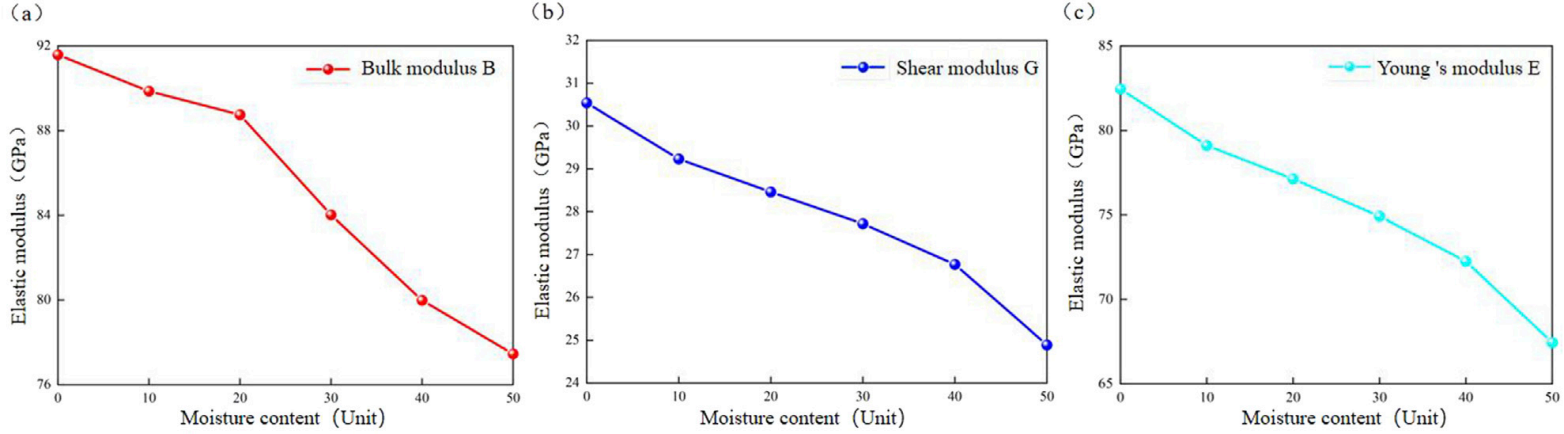
Reaction mechanism of elastic modulus of calcite to hydration degree. **(a)** Bulk modulus; **(b)** Shear modulus; **(c)** Young’s modulus.

Gradual hydration negatively impacts the bulk modulus, shear modulus, and Young’s modulus of calcite. As the degree of hydration increases, the modulus of elasticity decreases, and resistance to shear deformation diminishes (Figure 12; Table 9). The bulk modulus decreases from 91.58 GPa in the anhydrous calcite state to 77.46 GPa in the presence of 50 water molecules, indicating a decrease of 15.6% in strength. The shear modulus drops from 30.54 GPa to 24.89 GPa, reflecting an 18.5% reduction in strength. Similarly, the Young’s modulus falls from 82.45 GPa to 67.45 GPa, resulting in an 18.1% decrease in strength. The results are consistent with those of Ali et al. (2020), Zhang et al. (2023). Through literature investigation, it is found that with the hydration process of calcite, its interlayer structure expands, resulting in an increase in the diffusion coefficient of internal water molecules, thereby weakening the interaction force between atoms inside calcite. The increased hydration of calcite will reduce its elastic modulus and exacerbate the deterioration effect caused by hydration, which will adversely affect its overall mechanical properties (Jiamin, 2021; Chi et al., 2023; Wang et al., 2021).

**TABLE 9:** The bulk modulus B (GPa), shear modulus G (GPa), Young’s modulus E (GPa), Poisson’s ratio μ parameter of calcite under different hydration degree.

Number of water molecules (units)	Bulk modulus B (GPa)	Shear modulus G (GPa)	Young‘s modulus E (GPa)	Poisson’s ratio μ
0	91.58	30.54	82.45	0.350
10	89.86	29.23	79.11	0.353
20	88.75	28.46	77.13	0.355
30	84.02	27.72	74.92	0.351
40	79.98	26.77	72.25	0.349
50	77.46	24.89	67.45	0.355

## 4 Conclusion

The purpose of this study was to investigate the effects of temperature, pressure, and hydration on the microstructure and mechanical properties of calcite crystals. We started by building a calcite model. Then, the molecular dynamics simulation method was used to systematically analyze the changes of calcite microstructure and mechanical properties under different environmental conditions, and the following conclusions were obtained:

(1) When the temperature rises from 300 K to 1000 K, calcite crystal experiences noticeable expansion. The lattice parameters a, b, and c increase by 0.45%, 0.45%, and 0.44%, respectively. This leads to significant changes in the mechanical properties of calcite. The bulk modulus decreases from 91.58 GPa to 85.67 GPa, a reduction of 6.45%. The shear modulus decreased by 3.63% from 30.54 GPa to 29.43 GPa, and the Young’s modulus decreased by 3.92% from 82.45 GPa to 79.22 GPa.
(2) When the pressure increases from 0.1 GPa to 0.5 GPa, the calcite crystal shrinks. The lattice parameters a, b and c were reduced by 0.39%, 0.39% and 0.31%, respectively. The volume is also reduced by 1.10%. Under this condition, calcite exhibits higher hardness, greater deformation resistance, and improved ability to withstand volume deformation and shear failure. The bulk modulus increased from 91.58 GPa to 94.09 GPa, an increase of 2.74%. The shear modulus increased from 30.54 GPa to 33.40 GPa, an increase of 9.36%, while the Young‘s modulus increased from 82.45 GPa to 89.60 GPa, an increase of 8.66%.
(3) As the degree of hydration increases, the lattice parameters and crystal volume of calcite show an increasing trend, while the density decreases. The diffusion coefficient of water molecules in calcite is significantly enhanced. This enhancement facilitates the penetration of water from the surface into the interior, increasing the likelihood of structural damage to calcite.
(4) The mechanical constants of calcite decrease as hydration degree increases. The bulk modulus falls from 91.58 GPa in anhydrous calcite to 77.46 GPa in calcite with 50 water molecules. The strength decreases by 15.6%. The shear modulus decreases from 30.54 GPa to 24.89 GPa, with a strength reduction of 18.5%. Young’s modulus drops from 82.45 GPa to 67.45 GPa, resulting in an 18.1% decrease in strength.
(5) In this study, the microstructure and mechanical properties of calcite under different temperature, pressure, and hydration conditions were simulated, but there were still some limitations.

Limitations include:

a. The combined effects of temperature, pressure, and hydration conditions have not been comprehensively studied, leading to an incomplete understanding of their interactions as they occur in real-world geotechnical environments.
b. There is a lack of research on the long-term behavior of calcite under varying environmental conditions, which limits the assessment of its durability and reliability in practical applications such as tunnels, foundations, and underground storage.
c. The current simulation methods may not fully capture complex real-world conditions due to limitations in modeling accuracy and computational power, especially when applied to large-scale geomechanical systems with heterogeneous and dynamic boundary conditions.

Future research should focus on the following directions:

a. Carry out more systematic and integrated studies to explore the microstructure and mechanical properties of calcite under comprehensive and realistic field conditions, enhancing our ability to model calcite behavior in complex geotechnical settings.
b. Conduct long-term experimental and simulation studies on calcite’s performance in varied environmental scenarios typical of geotechnical engineering, such as cyclic loading, saturation changes, and chemical weathering, to evaluate its long-term stability and durability.
c. Utilize advanced computational simulation technologies—including multiscale modeling, coupled hydro-mechanical analysis, and AI-driven predictive tools—to predict the long-term evolution of calcite under field-relevant stress states and chemical concentration gradients, providing valuable insights for infrastructure design and risk assessment.

## Data Availability

The raw data supporting the conclusions of this article will be made available by the authors, without undue reservation.
