# Supplementary material for: Effects of temperature, pressure, and hydration on the microstructural characteristics and mechanical properties of calcite
Source: Front Chem. 2025 Jun 25;13:1568585. doi: 10.3389/fchem.2025.1568585 (PMC12239678; doi:10.3389/fchem.2025.1568585)
Supplement: Supplementary file 1 [file Presentation1.pdf]

Supporting Information

1 Simulation details

The simulation details are as follows: the long-range Coulomb force was calculated using Ewald's method with an accuracy of 0.0001, the time step was set to 1 fs (femtosecond), and the truncation radius was 12.5 Å. The classical equations of motion were solved using the Verlet integration method. The force field and charge parameters were selected from Dove's results. These results were obtained through calculations using density-functional theory and quantum chemistry. This approach provides high accuracy in understanding the complex interactions between atoms in calcite[1]. The force field is a mathematical framework that models the physical interactions between atomic particles. It defines how atoms bond, the angles between bonds, dihedral angles, and non-bonded interactions. By using a carefully chosen force field, we can accurately simulate the potential energy landscape of calcite. This allows us to capture the nuances of its microstructural behavior. To investigate the temperature and pressure effects on the microstructure and mechanical properties of calcite, molecular dynamics simulations under the NPT (Constant Number of Particles, Constant Pressure, and Constant Temperature) ensemble were conducted. The temperature and pressure were regulated using the Nose-Hoover method.

2 Calculation method

Changes in temperature, pressure, and hydration cause deformation effects in the crystal structure of calcite. These effects are primarily reflected in the coefficients of linear expansion and bulk expansion, the linear expansion coefficient (α) helps to predict how the material will expand or contract during heating or cooling. For many engineering applications, it is crucial to understand the linear expansion coefficient, because the inconsistency of thermal expansion between different materials may lead to stress concentration at the joint and even lead to structural failure. The volume expansion coefficient (γ) mainly reflects the stability of the material in high temperature environment. Excessive expansion may lead to component mismatch, thus affecting the overall performance and safety. Therefore, both the linear expansion coefficient and the volume expansion coefficient are important considerations in material selection and structural design[2], the calculation formula is shown in (1) ~ (2):

$$\alpha = \frac{\Delta L}{\mathrm{L} \times \Delta \mathrm{T}} \tag{1}$$

$$\gamma = \frac{\Delta V}{V \times \Delta T} \tag{2}$$

Where : ΔL is the given length change, L is the initial length; ΔV is the given volume change; V is the initial volume; ΔT is the change in temperature.

Molecular dynamics simulations were utilized to obtain the stiffness matrix and flexibility matrix of calcite in different environments. The

mechanical matrix is based on the mineral's intrinsic relationship and Hooke's law[3]. Hill[4]proposed an approximation method, considering the results of the Voigt and Reuss methods as the upper and lower limits of the true elastic modulus, respectively, and taking the average of the two as the elastic mechanical index of the material. The formulas for the bulk and shear moduli of the material were obtained according to the Voigt-Reuss-Hill (VRH) approximation method.

Bulk modulus (B_H) is a key parameter used to describe the volume change ability of materials under uniform pressure. It is defined as the ratio of the stress per unit volume to the resulting relative volume change, reflecting the ability of the material to resist compression. The higher bulk modulus indicates that the volume change of the material is not significant when it is subjected to external force, which is particularly important for the consideration of material strength and stability in the fields of civil engineering and geotechnical engineering.

On the other hand, the shear modulus (G_H) is used to describe the deformation ability of the material under the action of tangential stress. It is defined as the ratio of the tangential stress per unit area to the corresponding shear strain, which reflects the stiffness of the material under shear load. The higher shear modulus indicates that the material can maintain its shape and avoid large deformation when subjected to shear force, which is also of great significance in structural design and

material selection.

Through the analysis of bulk modulus and shear modulus, we can more fully understand the mechanical behavior of calcite under different environmental conditions, and provide a theoretical basis for effective engineering applications. The specific calculation formula is as follows (3) ~ (4):

$$B_H = \frac{B_v + B_R}{2} \tag{3}$$

$$G_H = \frac{G_v + G_R}{2} \tag{4}$$

Where: B_V is the Voigt bulk modulus, which represents the calculated bulk modulus under the assumption that the material is uniformly stressed in all directions. B_R is the Reuss bulk modulus, which represents the calculated bulk modulus when the material is assumed to be unevenly stressed in all directions. G_V is the Voigt shear modulus, which represents the calculated shear modulus under the assumption that the material is subjected to uniform shear force in all directions. G_R is the Reuss shear modulus, which represents the calculated shear modulus when the material is assumed to be subjected to uneven shear forces in all directions.

By calculating the B_H and G_H,, the Young's modulus (E) and Poisson's ratio (μ) of the material were determined, as shown in Formula (5) ~ (6) :

$$E = \frac{9B_H G_H}{3B_H + G_H} \tag{5}$$

$$\mu = \frac{3B_H - 2G_H}{2(3B_H + G_H)} \quad (6)$$

According to the theoretical formula, the modulus of elasticity of the calcite structure can be easily found. The modulus of elasticity measures the stiffness of a material, indicating the degree of deformation under a given stress. This property reflects the bonding strength between atoms in the crystal structure and determines the material's resistance to deformation. A higher modulus of elasticity signifies a stiffer material that is less likely to undergo elastic deformation.

The diffusion coefficient refers to the mutual collision between particles and the impact of particles on pore surfaces. It reflects the migration process of particles in space, indicating the relationship between changes in particle position over time. A larger diffusion coefficient corresponds to a faster diffusion speed. The diffusion coefficient evaluates the migration speed of water molecules in calcite with varying degrees of hydration, which is obtained using the Stokes-Einstein diffusion law[5], as detailed in Formulas (7) :

$$D = \lim_{t\to\infty} \frac{1}{6}\frac{1}{\mathrm{N_t}} \sum_{\mathrm{n=1}}^{\mathrm{N}} \left(|r(t) - r(0)|^2 \right) \quad (7)$$

Where: D is the diffusion coefficient; N_t is the number of statistical averaging time steps; t is the time, ps; N is the number of diffused atoms in the system; r(t) is the instantaneous displacement of the particle, Å ; r(0) is the original displacement of the particle, Å.

The mean square displacement (MSD) is the deviation of the particle

position relative to the reference position, and the reference position is the measurement of the particle activity level. The mean square displacement is measured over time to determine whether the particle has a diffusion behavior. The amount of mean square displacement of MSD corresponds to the diffusion coefficient of atoms. The MSD calculation formula is shown in (8):

$$\mathrm{MSD} = \langle |\mathrm{r}(\mathrm{t}) - \mathrm{r}(0)|^2 \rangle \tag{8}$$

Where: The sign ⟨ ⟩ is the average of all atoms in the group.

Reference

[1]Dove M T, Winkler B, Leslie M, Harris M J, Salje E K. A new interatomic potential model for calcite: applications to lattice dynamics studies, phase transition, and isotope fractionation. American Mineralogist. 1992; 77: 244-250.

[2]Pingquan W, Tao T, Junlin S, Qiurun W, Ping Y, Yang B. Review of application of molecular simulation in inhibiting surface hydration expansion of clay minerals. Chemistry and Technology of Fuels and Oils. 2022; 58: 63-76. DOI: 10.1007/s10553-022-01352-0

[3]Jia J, Wu D, Lin J, Jiang X. Molecular dynamics simulation of illite: From particle associations to hydration properties. Applied Clay Science. 2023; 234: 106850. DOI: 10.1016/j.clay.2023.106850.

[4]Hill R. The elastic behaviour of a crystalline aggregate. Proceedings of the Physical Society. Section A. 1952, 65(5): 349. DOI: 10.1088/0370-1298/65/5/307.

[5]Ahmad N, Adnan R, Soetens J C, Millot C. Molecular Dynamics simulations of liquid isoquinoline as a function of temperature. Chemical Physics. 2012; 407: 29-38. DOI: 10.1016/j.chemphys.2012.08.016.
